# Care for chronic illness in Australian general practice – focus groups of chronic disease self-help groups over 10 years: implications for chronic care systems reforms

**DOI:** 10.1186/1447-056X-8-1

**Published:** 2009-01-23

**Authors:** Carmel M Martin, Chris Peterson, Rowena Robinson, Joachim P Sturmberg

**Affiliations:** 1 Department of Family Medicine, Northern Ontario School of Medicine, London, Ontario, Canada; 2 School of Social Sciences, La Trobe University, Melbourne, Australia; 3 Australian Medical Association, Canberra, ACT, Australia; 4 Department of General Practice, Monash University, Australia; 5 Department of General Practice, The University of Newcastle, Newcastle, Australia

## Abstract

**Background:**

Chronic disease is a major global challenge. However, chronic illness and its care, when intruding into everyday life, has received less attention in Asia Pacific countries, including Australia, who are in the process of transitioning to chronic disease orientated health systems.

**Aim:**

The study aims to examine experiences of chronic illness before and after the introduction of Australian Medicare incentives for longer consultations and structured health assessments in general practice.

**Methods:**

Self-help groups around the conditions of diabetes, epilepsy, asthma and cancer identified key informants to participate in 4 disease specific focus groups. Audio taped transcripts of the focus groups were coded using grounded theory methodology. Key themes and lesser themes identified using a process of saturation until the study questions on needs and experiences of care were addressed. Thematic comparisons were made across the 2002/3 and 1992/3 focus groups.

**Findings:**

At times of chronic illness, there was need to find and then ensure access to 'the right GP'. The 'right GP or specialist' committed to an in-depth relationship of trust, personal rapport and understanding together with clinical and therapeutic competence. The 'right GP', the main specialist, the community nurse and the pharmacist were key providers, whose success depended on interprofessional communication. The need to trust and rely on care providers was balanced by the need for self-efficacy 'to be in control of disease and treatment' and 'to be your own case manager'. Changes in Medicare appeared to have little penetration into everyday perceptions of chronic illness burden or time and quality of GP care. Inequity of health system support for different disease groupings emerged. Diabetes, asthma and certain cancers, like breast cancer, had greater support, despite common experiences of disease burden, and a need for research and support programs.

**Conclusion:**

Core themes around chronic illness experience and care needs remained consistent over the 10 year period. Reforms did not appear to alleviate the burden of chronic illness across disease groups, yet some were more privileged than others. Thus in the future, chronic care reforms should build from greater understanding of the needs of people with chronic illness.

## Background

Chronic illness is when the disease intrudes upon everyday life in physical, psychological and social domains over a period of six months or more [1]. Chronic illness care for people has been conceptualised as ameliorating the burden of chronic disease as it impacts upon people's lives [2], while Chronic Disease Management focuses on disease outcomes and processes linked to disease – mostly primary secondary and tertiary prevention [3].

Depending upon a perspective, the terms chronic disease, condition and illness are either interchangeable, or convey different meanings. Most authors make philosophical distinctions between illness (and health) and disease. Disease strictly relates to a diagnosis based on a biomedical diagnostic classification system, while illness relates to the intrusion of unwanted and distressing symptoms and experiences into everyday life, related to diseases or conditions.

Complex Adaptive Chronic Care addresses multiple phases and stages of chronic illness and disease care [4]. It deals with multiple dimensions beyond single disease outcomes – bio-psycho-social and sense making or making sense of the disease(s), illness and care experiences [5]. This focus group study explores the sense making of people with chronic disease in the Australian context over time.

The global challenges of chronic disease prevention and the management of chronic illness are international. In 2005, there were predicted to be 35 million deaths from chronic diseases [6]. It is a global concern that has considerable urgency in terms of impact on population well being and economic burden [7]. Common concerns have been identified across developed, developing and transitional countries [8] and the increased ageing of the population exacerbates the problem. The Asia Pacific region has particular concerns arising from the rapid health transition to obesity and diabetes [9].

There have been considerable efforts to implement chronic illness care through a number of initiatives through general practice/family medicine/primary care in the past 10–15 years both internationally [10-15] and in Australia [16-18]. Australian initiatives include Enhanced Primary Care items such as Care Planning and Case Conferencing [19], Co-ordinated Care Trials [17], Sharing Care (self-management) [20] and more recently the National Chronic Disease Strategy [21] around specific diseases in the National Health Priorities [20]. Other Asia Pacific countries such as Singapore and Malaysia are introducing Chronic Care Model style initiatives [22,23], and national chronic disease networks are being set up in China and India.

There has been a slow uptake in Australia of the key components of what is now known internationally as the Chronic Care Model [11]. For example, a recent qualitative study of GP perceptions of chronic illness care indicated that such initiatives may have had a minimal impact upon their everyday practice [24]. In Australia this has been attributed to constraints of time and a balance of responsibilities [24]. As Harris stated in November 2008, *'the capacity of general practice to take these *(components of the chronic care model) *up has been constrained by funding and workforce availability' *and that he supports the more intensive major workforce and health system restructuring planned by federal and state governments [20]. In addition, a recent 2008 Commonwealth Fund survey of people with complex chronic care needs supports the need for ongoing improvement of chronic illness care in 8 developed countries [25].

On the other hand, there is international evidence to challenge the effectiveness and efficiency of many current approaches towards restructuring of health care to introduce major aspects of the Chronic Care Model [26,27]. The Chronic Care Model appears to be better suited to prevention of disease, than cost effective management of chronic illness [3,17]. Linking the Chronic Care Model to incentive based payments as in the UK, appears to deliver improved performance on certain chronic disease parameters [28]. However, performance was improving at a rapid rate based on better information and practice organisation before the introduction of incentives [29]. Alternative models of narrative based medicine [30], transformative relationship based medicine [31] and complexity science [13,32] may be more appropriate to address the need to transform health systems to meet the needs of those with chronic illness, rather than the top down linear and implementation 'science' based enforcement of rigid protocols which emphasise the disease rather than the illness [4,28,33]. Other models of system transformation such as complex adaptive chronic care are dynamic, adaptive and bottom up building on individual needs in chronic illness [14,33,34]. Complex adaptive chronic care recognises different stages in the chronic condition trajectory with different strategies and system reform approaches needed to address the journey from primary prevention to chronic illness to pre-terminal care. In this context, our study qualitatively explored the illness experience and care experiences of Australian people with chronic disease. Focus groups of key informants who were members of self-help groups and who were willing to share their stories of having chronic illness and providing peer support for self-management. This study compared their experiences in 2002/3 and 1992/3 [35].

## Aims

The study aims *to investigate the common and differing experiences of important chronic disease groups over time in relation to their experiences of illness in the context of health system reform*. This study aims to follow up previous work undertaken by the chief investigator, approximately 10 years after the initial study, examining patients' perceptions of chronic illness care provided by general practitioners, primary care and the health system more broadly [35]. *This study aims to inform other countries, as well as Australia, who are in the process of transitioning to chronic disease orientated health systems*.

## Materials and methods

Focus groups were conducted with four self-help/consumer groups representing patients affected by epilepsy, asthma, cancer and Type 2 Diabetes in the ACT region, replicating and adapting a previous study [35]. The groups were conducted using theoretical sampling; that is they were conducted until no new themes emerged and there was thematic saturation with no new variations emerging. Rice and Ezzy [36] recommend the use of focus groups to gain insights into personal experience and to refine and develop ideas about common experiences. The methodology is a grounded theory approach based on that refined by Glasser and Straus [37]. This approach was used by the chief investigator in her previous research with chronically ill patients.

### Participant Recruitment

In the original research four focus groups were undertaken with members of self-help groups from the following organisations – Epilepsy Association of the ACT, the ACT Cancer Society Asthma Association of the ACT and the non-insulin dependent Diabetes self-help group of Diabetes Australia, Canberra. These four groups were invited again to participate in the current research, and with the exception of the ACT Cancer Society (renamed Council) all agreed to participate. The ACT Cancer Council was already engaged in participating in other research; however they suggested another local cancer support group, Bosom Buddies, who agreed to participate.

Each of these groups recruited participants from their membership. Not all groups had active self-help groups at the time. A total of 32 people participated with 10 from Epilepsy ACT; 5 from Asthma Association; 6 from Bosom Buddies and 11 from Diabetes ACT. Signed consent was obtained from each participant. This study was approved by the Royal Australian College of General Practitioners Research Ethics Committee.

It is recognised that this method produces a sampling bias but it did provide the most effective convenience sample for the project, and ensured a range of comparative answers to the researchers' questions. In addition, as key informants, ACT self-help groups are largely comprised of the highly educated middle class of Australian society with many public servants or ex public servants residing in the nation's capital. They are articulate and empowered and are likely to be early adopters of innovations and improvements in their care.

### Analysis

Each focus group lasted between two and two and half hours and was audio taped and transcribed. Rice and Ezzy [36] recommend this method for gaining a good spread of ideas form study participants. The project officer and chief investigator independently categorised key themes, in an exploratory and open ended manner, of the first focus group (Epilepsy ACT). The themes and categories coded independently from this transcript were then analysed by the project officer and chief investigator together to clarify commonalities, differences, outliers or emergent phenomena. This was also checked back to the original focus group categories and codes derived by the use of grounded theory, iteratively to develop a framework for analysis. Coding was then carried out independently by the two researchers and the same process repeated until saturation and crystallisation of themes was achieved. For subsequent groups the project officer and chief investigator each independently coded half the transcript prior to discussion and clarification of the complete coding. Discussion of the coding and results were undertaken by face to face and teleconference meetings with the co-investigator.

A matrix was developed to allow a comparison of the themes from the current groups with those of the original research. Emergent themes were identified. In the writing of the report key themes were aggregated into broad issues to address the research questions. The final draft reports were sent to the self-help group members for member checking. The Epilepsy self-help group in particular felt 'understood' by the focus group study.

## Findings

To contextualise the findings of this study we briefly summarise the characteristics of the illness and care experiences and the insights gained from the original focus groups in 1992/93.

### Original Focus groups 1993

In 1993, the self-help group members who attended the focus groups described themselves as core members who were involved in the running of the organisation or were in a crisis period as a result of their disease status.

The key themes from the original groups related to the qualities desired in, and the concept of, the 'right GP'. Qualities of the 'right GP' included good disease knowledge and diagnostic skills; commitment, caring and understanding and a trusting and therapeutic relationship. The original groups also identified issues around the lack of time in GP consultations for chronic illness management and the need for appropriate access to, and availability of, GPs and other health professionals. Appendix 1 outlines the key issues in the initial focus groups in more detail.

### Follow up focus groups

We first described the participants' experiences of 'being a patient with a chronic disease' and their common experiences and disease-specific management experiences in general practice. In the second part we outline the remaining study questions regarding how the GP might better assist; examples of successful and less successful chronic illness care; and how chronic illness care in general practice changed over the last 10 years.

#### Epilepsy Group 2003

The Epilepsy Group participants were associated with the organisation but not part of a formal support group. They were convened on an ad hoc basis. Participants ages ranged from a primary school aged child to an aged pensioner and there was an even gender split amongst participants.

Difficult to control epilepsy was devastating. '*I was so psychologically destabilised by the grief of losing who I was *(and) *then coming to grips with the degenerating condition'*.

Most participants experienced that their GP did not know enough about epilepsy and its medication to be able to provide management and support for it. Comments such as '*they don't know anything, so they can't help me*' typified this. With drug therapy a central aspect of epilepsy care, most care was provided by the neurologist if not an epilepsy specialist. Numerous issues were raised around referral to specialists including whether referral should be to a neurologist or epilepsy specialist. Finding the right epilepsy specialist or neurologist was of paramount importance as there was a general consensus, that for people with challenging epilepsy, the right biomedical therapeutic was critical. The self-help group networks allowed informal 'insider' comparisons of GPs and specialists and provided advice on who to attend when care wasn't working. *'Wouldn't attend him in a million years' *was a common verdict about a certain specialist. *'My GP's OK, I trust him because he has served me and my father well, although he doesn't know anything about epilepsy. At least he knows what he doesn't know, so I trust him if I have the flu or other sorts of problems.'*

Issues regarding medication were prominent, particularly relating to side effects, the years spent trying to find the right medication and the impact on quality of life with one person when speaking of experiencing *'the shakes' *as a side effect of a particular medication saying, '*I preferred to have the seizures*'. To participants, epilepsy was not a priority to medical research, the community or government – there was a feeling of '*take your pills and shut up*'. Loss of jobs, licences, control during fits and friends were pertinent and distressing. Experiences resulting from community stigma and lack of understanding and knowledge of epilepsy are exemplified by. '*Once they know you have epilepsy and still have fits, they are frightened to employ you on manual jobs*.' and '*We keep our epilepsy hidden ... as much as possible.'*

#### Asthma Group 2003

Asthma group participants were associated with the organisation rather than belonging to a self-help group as such. Most were female and aged over 40 years. Several, including Ben had a long standing relationship with the organisation and were advocates for better care. Ben described his experiences and felt he had '*lived on the knife edge for 20 years with severe asthma and COPD, and an eye condition called glaucoma*'. He was desperately afraid of having a bad attack of asthma and landing in an emergency department somewhere and being given injectable corticosteroids which could render him blind. '*My GP and asthma specialist know me and I am desperate to go through them and get directed into care ... I am working very hard to try to make sure that doctors know about asthma.'*

Participants felt that some GPs had a good general knowledge regarding asthma and its medications. Most felt that their GP referred and consulted appropriately with their specialist. The importance of good communication with the GP was emphasised by the group as well as the need for a doctor with holistic skills to care for chronic illness. Several spoke of the fear involved in a serious asthma attack, particularly if it was in relation to their children. *'Fear and panic can be overwhelming.' *Sometimes it is difficult to be taken seriously by medical practitioners. '*It can be very hard to get past receptionists at time. They just fob you off to emergency.' *Most spoke of the chronicity of asthma and the ongoing efforts and battles involved in keeping their asthma and other lung conditions particularly chronic obstructive lung disease well controlled, trying to prevent crises and to live a normal life. Alice had been plagued with exacerbations of her long term lung problems. This culminated in a recent hospital admission a few weeks ago. Since her discharge from hospital, Alice claims she *'has not been the best'*. Despite this, she has continued to keep up with her many social activities. She '*never gives up'*.

#### Cancer Group 2003

Most of the cancer self-help group – 'Bosom Buddies' – focus group participants had had a diagnosis of breast cancer. One woman was experiencing a recurrence of cancer at the time of the meeting and one participant was the husband of a woman called June who had died from brain cancer. June had no access to a brain cancer support group throughout a gruelling battle to her death. She and her husband, Brian, had been 'adopted' by Bosom Buddies. All were over 40 years of age.

The issue of finding the 'right GP' was a particularly key theme in the cancer group. Also, the need for good communication with the GP and between the GP and the sometimes multiple specialists was emphasised. '*Having a GP who listened to and treated concerns promptly and appropriately*;' was paramount in the ever vigilant defence against the constant spectre of potential recurrence. Thoroughness and a responding to 'gut' feelings of the patient were recurrent themes and key qualities desired in a GP, for example, '*I knew it was a recurrence but I had a hard time convincing people of that.' *This was particularly important in relation to diagnostic skills and looking at a person holistically rather than focussing on the results of a particular blood test or scan. There was a need to '*listen to the whole body.'*

#### Diabetes Group 2003

All the focus group participants had mature onset diabetes ranging from those controlled by diet and exercise through to medication. They were all members of the Diabetes Australia support groups and met regularly for activities and support. There was a fairly even gender split with most participants being of retirement age. One of the members, Joan, was a participant in both 1993 and 2003 focus groups. She was recovering from her first heart attack. However she was grateful for the developments in diabetes care and felt very secure. *'I just thought of it as being inconvenient in some ways.'*

A strong theme for participants was that diabetes care and management was their responsibility. *'But really the responsibility is our own because he can't cure the disease, he can manage it and that's what we've got to do, just manage it' *and *'apart from smoking and smoking related diseases, it's probably the most patient controlled illness'*. There was an almost mixed reaction to the advent of HbA1c testing that gives a reading on the degree of blood sugar control over the previous three months with some patients feeling a loss of personal freedom and omnipotent control by the doctor as is illustrate by this quote, *'you can play up for (just one week) and get into trouble'*.

For most, their GP managed their diabetes with only a few consulting a specialist. The issue of learning and the need for accurate information was heavily emphasised, for example, *'that's what diabetes is, it's always learning, always learning'*.

## Key themes to emerge from the research

The main themes focussed on general practice access and availability; health system issues; disease issues and other issues. Focus group themes were identified and categorised. Strong themes were expressed as being important by more than half the group and reached saturation early. They were also spontaneous without prompting. Moderate themes were presented often resulting from interviewer prompts. Themes that were not present were neither spontaneously stated nor stated in response to interviewer prompts. Key findings are shown below in the (Appendix 2) and reported in more detail below.

### General Practice Access and Availability

One participant summed up the challenges of access and availability as threefold in '*finding a doctor and getting in and having enough time'*.

#### Finding a GP

Many in the groups reflected current concerns regarding the availability of GPs. Several had experienced the difficulty of finding a new GP when their GP retired or closed the practice. Many had either personal experience or were aware of situations where GPs had 'closed their books'. Several spoke of the relief of having found a GP they were satisfied with and having become a patient before the *'books' *had been closed.

#### Finding the 'Right GP'

One of the main recurrent themes of the original and current research was finding the 'right GP' in terms of professional and personal qualities. Sometimes finding the 'right GP' takes on a sense of urgency, '*When you are younger and well and the kids just have minor illnesses, it doesn't matter so much *... *then all of a sudden to jump into this world of malignancy *(cancer), *you're in a different ball game ... Well what we did once it became serious, we looked for a good GP and that was important in the whole process, a sympathetic GP and we were very lucky to get associated with him'*.

Some noted the importance of the GP when facing chronic illness, for example, *'chronic disease, that's when you need your time, your space, the knowledge, the experience and the skilled GP'*. One person commented that the 'right' was a time influenced event in that '*the GP you've got today might not be the one you need in five years time when you condition's different'*.

Finding the 'right GP' is a process of finding the one with the desirable qualities. How is this done? Participants commented on the lack of guidance for people in this process and how it often came down to *'word of mouth' *although this was obviously difficult for those new to a city. Self-help groups provided considerable support when people with serious need were new to a city and looking for a GP. For many participants the length of relationship they had with their GP was a significant aspect in the ongoing trust, confidence and personal rapport. They gained a lot from this continuity of care. '*He instils confidence *... *He gives me the impression – he seems to have a common sense approach*... *No, he *(has been visiting this patient for over 20 years) ... *he got me through that *(exacerbation of condition) *amazingly ... he was brilliant. Three months it took, and together we will do it, and that is his approach.'*

### Qualities of the 'Right GP'

#### Supportive, understanding and a good communicator

Focus group participants were consistent in the qualities they were looking for from their GP and reaffirmed the qualities identified in the original focus groups. Understanding and supportive, trustworthy, holistic care, good listening and communication skills were key qualities participants were looking for. '*They're looking at the whole picture'*. These qualities were important even if their specific medical chronic illness care came from their specialist. Other qualities included being interested, thorough, and approachable; having a good sense of humour, empathy, and being able to talk to about concerns. It was vital to recognize the emotional state of people at the diagnostic or transitional stages of disease as is exemplified by '(you) *only hear *(the) *first bit of information and after that you're in shock*" and '*She understood that I was in shock and rang me later at home ...'*

It was important to patients to be seen as a whole person, for example *'she sees me as a diabetic person rather than as 'a patient'' *and *' ... treat me more as a personal patient ... an individual ... rather than just someone who has epilepsy'*.

Matching of expectations was another important quality. '*A distinction has to be made between patient and general practitioner expectations of the interaction, which may differ markedly and would seem to require a 'match' between the general practitioner and the patient in order to obtain mutually satisfying outcomes of the interaction during *(the) *consultation.'*

#### Disease knowledge

Most groups identified that there was a general lack of knowledge amongst GPs of their particular condition, its treatment and medications. The GP was however regarded as a key source of general information. Whilst recognising GP heavy workloads and the fact that they were not specialists, all groups identified the importance of GPs keeping up to date with latest information particularly in relation to medications. '*We are in their hands and we are vulnerable.'*

#### Diagnostic skills

GPs diagnostic skills were regarded as extremely important whether in making the original diagnosis of the chronic condition or in relation to aspects of other conditions. Some comments emphasised the difficulty in the GP role in being able to discern between the trivial and the serious of symptoms. Others noted the importance of generalism in diagnosis and management *'I suspect too that the true value of a GP is just that, general, everything'*.

#### Referral to specialist care

Referral issues were prominent in most groups. For patients with epilepsy there was the issue of being referred to a specialist with sufficient specialised knowledge while for those with multiple specialist involved, for example with cancer care, it was important to have good links between all the specialists and the GP.

Whilst many participants assumed there was ongoing verbal communication between their GP and specialist few were able to confirm this. However many were happy with the links between their GP and specialist with one person commenting '*between the doctor and the specialist, they seem to work in tandem'*.

#### Prescriptions

With the exception of the cancer group, participants in the other groups identified the provision of prescriptions as a key reason for attending the GP. For some this was their prime reason as particularly with the epilepsy groups they did not perceive the GP as having much of a role in their disease care. Pharmacists had an important role in monitoring and supporting medication use, particularly in epilepsy when most of the prescribing is by specialists and GPs are limited to writing repeat prescriptions. '... *it's as if he *(the GP) *didn't know because I just went through a whole heap of pills*' from the specialist that didn't work and he (the GP) just repeated the prescriptions.

### Access to the GP

#### Difficulties of getting an appointment

Some noted the increasing difficulty of getting an appointment at short notice *'it's like, if you're very unwell you just have to sit unwell and just cope as well as you can until you get access to the doctor'*.

In most groups there was some discussion of locum medical practitioners. There was general dissatisfaction regarding the thoroughness and quality of care from locums. Several had had experiences of being given, or nearly being given, the wrong medication with others experiencing their symptoms as being trivialised and resulting in a reluctance to see a locum again. '*I will wait until my GP returns. The locums are generally useless.'*

#### Consultation length

The general experience across the four groups was of not having sufficient time in consultation with the GP. One person noted: '*Sometimes you actually just want to have a big talk just for... reassurance maybe ... and that is the time that you are feeling ... the need ... it is so disappointing when you are pushed out the door'*. Some participants in most groups had experience with requesting longer consultations, at the time of making the appointment, and found this valuable. Another person commented: *'My feeling is that most GPs are so busy and their time is so precious. And they make it that way, I mean; they don't have to.. in my opinion. They could decide to spend 25 minutes with every patient and to hell with whatever the statutory rate is and just make less money but still have, I would have thought, a more interesting life'*.

Many participants felt that GP's had been increasingly busy and were concerned that some government initiatives, whilst good ideas, would add further to this burden. In relation to the introduction of care planning and the chronic disease items one participant noted, '*And I just wonder how they are going to fit more in'*.

#### Other Providers and Care in the home

This study focussed upon GP care; however other providers were identified as playing key roles.

Community nurses were seen as especially important, *'Community nurses have holistic view and ability to refer on'*. and *'They are wonderful'*. They have a central role in the continuity of care in serious sickness and disability. Palliative care nurses and palliative care doctors were so important in the terminal stage of home care, as their regular GP did not visit. *'Oh – the GP he never came to the home*. *We relied on the home-based palliative care, community nurses, specialists, hospice in that situation.' *Yet, coordination of care was challenging.' *One could only trust they were all travelling in the same direction.' *Pharmacists were particularly helpful in stages of care where medication was a major feature of the therapy, especially when there were frequent dosage and treatment changes, and sometimes provided considerable informational and at times relationship support. '*I really rely on my local pharmacist. We are good friends over the years.'*

### Health system issues

#### Enhanced Primary Care/Structured Care

On average, only one person in each group was aware of the Enhanced Primary Care or chronic disease initiatives *per se*. Most seemed to feel that care planning [Appendix 3] [38] was a worthwhile initiative that could help organise their care better. Some groups expressed concern about the fairness of GP payments for case conferences [Appendix 4] as this was regarded as something they should already be doing. When asked about changes in primary care over the last 10 years most felt there had been little, if any improvement. '*I don't think it has changed much, everyone seems to be busier and no one has time to talk and explain.'*

The Enhanced Primary Care items were designed to deliver more coordinated, structured care to patients, however, based on this sample there was little experience of their penetration to everyday care.

A subtle change in language had emerged over the 10 year period. There were examples of appreciation of the use of structured care in terms of three monthly diabetes checks, use of Information Technology (IT) systems. Some comments were made on the role of the GP as 'case manager'. Many made reference to desiring that their GPs would be able to access hospital records electronically. *'I would so welcome my GP getting my hospital records so that she could understand and explain to me what happened there. It is like a black hole, my experiences there.' *and *'We know there are privacy issues, but I want to understand what happened to me, I want to understand what happened to my body*.' While being cognisant of the impact of privacy concerns on the implementation of IT, several participants noted the used of IT systems for various aspects of the consultation and felt that informational continuity that they provided was very important in their chronic care.

#### Other initiatives

Only one person was aware of, and participating in the Sharing Health Care [Appendix 5] (self management program) and this person spoke positively, regarding it as '*really helpful*'. Others in the group noted the barriers to access to this program that was restricted by age and having certain conditions.

Most in the asthma group were aware of the Asthma Management Plan Program with several raising concerns that the program delivered payment to doctors for better management of their asthma rather than patients who did the work – '*the incentives in medicine, in health care, are focussed on the professionals as opposed to some of the incentives being within the community or the consumers*'. [Appendix 6 describes the modification of the program discussed at the focus groups which still pays the practice rather than patients for good care]. Whilst only one person was aware of the GP yearly payment for a diabetes register and structured care, many were engaged in regular three monthly diabetes checkups but were not aware of these as specific government initiatives.

#### Change over time

Many participants experienced their GPs as being very pressured these days, for example '*There's more pressure on doctors now days ... I really feel when I go to see him that he's on edge*'. Participants in some groups noted the improvement in medications or management of their condition over time.

One person noted their changed experience of general practice care as a trajectory moving from a shopfront type of practice to a group practice to one now where he has an ongoing relationship with his GP. Others felt their care had become more impersonal and mechanical.

#### Prioritisation of Issues

An issue was raised in regards to government prioritisation of issues and concentrating on areas where they perceive they can make gains to the detriment of other conditions. For example, '*having been associated with one of the minority areas I know what it's like from the outside, seeing a lot of work done, a lot of attention, a lot of focus, a lot of booklets, resources, support groups for some conditions such as breast cancer compared to fumbling around in the dark, struggling, trying to find someone to make some sense of this very minority type of grouping you're in – brain cancer'*. Whilst not made by a participant in the epilepsy group it has particular resonance from the participants in that group and the lack of knowledge, research and resources related to epilepsy.

### Disease issues

#### Control

The issue of control was a recurrent theme throughout the groups although the context varied. For some it was control of quality of life or medication side effects while for others, as in the case, of asthma, it was of the condition, for example '*the fear that's behind the scenes when you do lose control' *and *'It takes at least a month frequently to get back to the level of control I had before that exacerbation.'*

#### Self-help organisations

Almost all participants spoke very positively about the benefits they gained from involvement in the self-help organisation which had provided significant assistance in the support and education aspects of managing and coming to terms with the condition. Several noted the importance of education and support for family members. However, *'not everyone is a support groups participant' *by nature. It was interesting to note that very few people were referred to the self-help organisation by their GP. One person also noted the support that was available through Internet based groups, particularly for 'smaller' cancers with less prominence. Peer support was also very important.

#### Self-education

The importance of learning and educating oneself about the condition was highlighted by all the groups. The role of the Internet in facilitating this was touched on in several of the groups. Whilst self-education was still regarded as a key issue in the epilepsy groups it also highlighted the relative lack of available information compared with some of the other conditions.

#### Self-management programs

Self-management training and support were perceived to be useful, but restricted to specific age groups, locations and types of diseases. The common overriding themes about the experience of serious illness were fear and anxiety, the need to trust medical care providers balanced by the need to make your own way and 'to *be your own case manager'*. Knowing about the next steps and *'the nature of the beast' *and how to manage the disease and its mental and emotional concomitants for the sufferer and the carer were huge existing gaps. Training of GPs and all health professionals in this regard would be invaluable.

Common themes – narratives of experiences of health and illness differed across diseases according to the predominant pattern of bodily dysfunction, yet fear and anxiety and a shaken trust in their own body were ubiquitous. Gaining *control *of the condition was the most important goal in disease management. Control was secondary to cure, which was the ideal. Compared to the initial focus groups, the later groups had perceptions of considerable improvement in medical knowledge for asthma and diabetes. Cancer was a variable field with perceptions that some cancer such as breast and bowel were much advanced while other cancers such as brain cancers were neglected. Epilepsy was perceived to be the least well researched and understood across the whole spectrum of the conditions. Community lack of knowledge of disease issues was perceived to be lacking with associated stigma and discrimination and this was noted in relation to epilepsy and some cancers.

## Discussion

Chronic disease management has attracted a lot of attention in the Australian and international context because of its impact on mortality, health care resources and its economic burden on governments and health care funders [6]. In an effort to ameliorate these threats and deficiencies the Australian government has spent many millions of dollars on Enhanced Primary Care initiatives and a National Chronic Disease Strategy. In Victoria, Australia's second most populous State, an initiative called Primary Care Partnerships have been an attempt to link local government initiatives with both the State government and with private general practitioners, pharmacists and community nursing services in their area to ensure the development of relevant care coordination and case management structures for chronic illness. Other States and Territories have had similar programs, which are all essentially top down, building on government and health professional views of care needs. Much less attention has been paid to the experiences of people with chronic disease in the health care system, particularly in primary care. To what extent are these initiatives responsive to chronic illness and how has general practice and primary care reform change impacted on people's experiences of care?

While these focus groups are of key informants from chronic disease self-help groups from one locality, Canberra in the Australian Capital Territory, they typify a wide group of patients who described themselves as educated, informed and articulate. Some members were leading in policy making around patient support initiatives and likely to be well informed, comfortable with their condition and its social situation. They were prepared to be actively involved with the professionals and support groups, whereas other members described themselves as having difficulty with their condition, difficulty gaining and maintaining employment, and feeling socially marginalized.

These observations reiterate the need to consider psychosocial aspects in the management of chronic illness to be equally important as disease-specific treatment. As these informant groups highlight one can successfully adapt to ones lasting disease and optimize the experience of 'good health' whereas for others their ongoing problems impact continuously and negatively on several aspects of their life. An issue not often recognised was the social response to chronic disease – stigmatisation of chronic disease was particularly noted in relation to epilepsy and some cancers [39], with diabetes and obesity and smoking related disorders, being conditions where there was, perhaps, increasing stigmatisation [40].

From a philosophical as well as a pragmatic perspective chronic disease management must reflect the needs and values of the individual patient. As a consequence chronic disease management programmes must provide flexible approaches in a resource sufficient environment, and all collaborators in a chronic disease management programme need to accept that they are not always needed all of the time.

It is therefore not surprising that this group very powerfully expressed the need for an in depth personal therapeutic relationship with a primary care physician, reaffirming the needs expressed in 1992/3. The 'right GP' plays the role of healer, confidant and scientific expert with the self-knowledge and courage to admit the limits of their knowledge. These findings are encouraging for the educators and planners of general practice. They delineate an interconnecting adaptive (generalist) GP role in chronic illness, with in depth skills in diagnosis, treatment, active listening and therapeutic relationships across disease and illness. Ongoing reform needs to strengthen person-centred primary care approaches in order to better address both intense threatening illness and disease-based dynamic changing needs of the ill and vulnerable at low ebb in their life course.

The fact that self-help group members included highly educated and politically aware members who were almost completely unaware of the enhanced primary care initiatives is challenging. This is particularly perplexing as these participants were strongly engaged for many years prior to the Australian government initiatives in an 'expert patient' philosophy [41]. Perhaps there merely reflects a slow diffusion of the initiatives of the Chronic Disease Strategy and Enhanced Primary Care, and it may have changed even to this date. On the other hand, it may reflect an Australian chronic disease program that is still conceptualised as a complicated top down rather than as a complex bottom up system change.

Given the self-management and self-care aspects of chronic disease, many participants indicated they may have chosen to avail themselves of particular Australian initiatives – particularly multi-disciplinary care planning. However, the general theme was that GPs were getting busier and would not have time to sufficiently participate. Of concern is the perception that the Enhanced Primary Care and Chronic Disease programmes seem to be designed by bureaucrats for accountability purposes, based on what other countries were doing, rather than trying to help the GP in their care.

Clearly there is a need for further studies to fully understand the poor penetration of enhanced primary care initiatives to assist people with chronic disease. A recent analysis has confirmed the slow uptake of initiatives, perhaps limited by GPs capacity to absorb the extra work into their every day practices [20]. In addition, access to consultation time is perceived to remain under pressure, perhaps greater than ever. Our qualitative findings are in line with those in other locations as reported by Infante et al [42]. While the language is slightly different, the relationships and social interactions in chronic disease care were found to be similarly important [42].

Healing is an important concept and therapeutic relationships that are attuned to the patient narratives [43] are an often unrecognised aspect of the Chronic Care Model [31,44].

Are these findings relevant to developing countries and non Western health systems?

The international literature supports the universality of the burden of chronic disease. Control is understood to entail a shift to a Western individualistic model of health with the aim of managing disease. Studies of women in Philippines, Thailand, Malaysia, Canada, Hong Kong, and Singapore have identified common factors in the need for greater medical understanding of disease [8].

Yet, globally, it is health and well being which is arguably more important. The WHO Commission on Social Determinants of Health defines health as *'the extent to which an individual, family or community is able to realize aspirations & satisfy needs to cope with their environment' *[45]. There are many definitions of health which have major commonalities all stressing patient centred care around the personal experiences and the need to reconstruct the self in the light of chronic illness, despite cultural differences [22,46]. While the notion of the patient as 'expert' is not a traditional approach of most Asia pacific countries, peer support for self-management may well fit into the socio-cultural framework. Also, despite commonly accepted differences in Western individual orientation and non-Western collective social orientation, a bi-culture is emerging in Asia with a blurring of individual and collective responsibilities [47].

In regards to research, epilepsy was perceived to be very poorly supported during the past 10 years. People with asthma and diabetes in contrast felt that therapeutics had improved their support. Cancer was divided into the common 'popular' cancers such as breast cancer which received a great deal of government funding and support for sufferers and rarer cancers which were the "Cinderellas" of cancers. The prioritisation of some chronic disease and illness over others is clearly not based on social justice concerns and equity. Rather it is based upon perceived public health and economic burden [6].

However, that does not mean that results can be immediately generalised as evidence for health system performance in different countries?

Cultural differences among patients and contextual differences in health systems are very entrenched and important considerations. For example, Australian patients have a highly developed 'consumer awareness' having a history of being able to shop around for the care they 'want' as distinct from 'need' and have a Western individualistic culture. In contrast, patients in other countries have had little choice and have passive acquiescence to what the health system has to offer.

Similarly many constructs examined in the survey, such as 'access', differ in various contexts. Other areas that need closer analysis and examination of underlying assumptions include the uncritical use of the Chronic Care Model as a service template rather than as a conceptual framework for chronic care. However, this focus group study is a barometer of what is happening in chronic illness care and indicates the importance of building change and improvement on local culture and existing system contexts and successes based on in depth analysis of existing strengths as well as apparent short comings. Such qualitative studies as this are the personal stories and experiences to accompany other indicators of complex care [25] and as such as are a warning light that draw our attention to areas of concern. Indicators need detailed and triangulated studies, often additional in depth research and very careful interpretation, before they are evidence for policy change over time.

Understanding generic chronic care in complex health systems requires us to observe and understand the lived experience and sense making of those who go through the care, as much as about the disease and its management.

## Conclusions and recommendations

Chronic illness is a major social phenomenon as well as a biomedical and economic challenge, and occurs when the disease or condition intrudes on people's lives and personal experiences. In the Australian context making sense of chronic illness experiences through personal relationship based care with the 'right GP' or right specialist and other professionals, were central to better experiences of illness.

Gaining control of the sense of self and holding on to one's identity was a strongly emergent and spontaneous theme across conditions and time. Self-knowledge of one's own disease and body was universally agreed to as being the most important prerequisite to gaining control.

The main themes identified in response to the study questions were the importance of a person-centred and technically competent general practitioner and did not substantially differ from the 1992/3 focus groups. The intensity of need for the 'right GP' when people were in a phase of serious chronic illness was reaffirmed. An in-depth relationship of trust based upon personal rapport and understanding and clinical and therapeutic competence with the ability and commitment to refer to and liaise with specialists and other professionals was a strongly emergent theme.

The self-help group members had little experience of the enhanced primary care initiatives. Care planning was the initiative that was identified as being potentially most helpful in general practice care. Self-management programs that linked with the self-help group activities were perceived as potentially helpful. However, how they are introduced needs careful planning and it may be that a variety of self-management programs offering choice to patients may be most desirable. The need for case management, support and self-management was a key feature when the illness was complex and patients were most ill and vulnerable. Government prioritisation and competition for worthiness and support impacted upon people's experiences of care. This need has been recognised by many initiatives and trials, but as stated by Harris in 2008 and others previously – these initiatives have been very slow to become mainstream activities [18,20,48].

Yet there are many complex issues to deal with in health care reforms, and one has to be particularly wary about the temptation of 'one-size fit all' solutions. Implementing the Chronic Care Model, which itself has shown a number of significant deficiencies, would require a major restructuring of health services. It is important that the 'wholesale' introduction of changes such as self-management and lifestyle change does not interfere with relationship based care and those aspects that are already working well in the current system when patients in the phases of serious chronic illness are most vulnerable.

## Competing interests

The authors declare that they have no competing interests.

## Authors' contributions

CMM conceptualized, designed, achieved funding, and lead the focus groups their analysis and write up. She conceptualized, designed, achieved funding, and led the initial focus groups, their analysis and write up under the supervision of Dr Jeanne Daly during her PhD studies. RR managed the project, the organization of focus groups, the data collection and audio taping; she conducted the initial analysis of the focus groups and with CMM conducted the thematic analysis and the write up. CP, Co-Investigator, provided valuable sociological expertise to the funding application and the methodological conduct of the study. He provided a third analytical perspective to the thematic analysis conducted by RR and CMM to ensure validity. He made a significant contribution to the write up of the material. JPS contributed to the analysis and interpretation and write up of the study.

## About the authors

Carmel M Martin, MBBS, MSc, PhD, MRCGP, FRACGP, FAFPHM is Associate Professor of Family Medicine at the Northern Ontario School of Medicine, Canada

Chris Peterson, Ph D. is Lecturer at the School of Social Sciences, La Trobe University, Australia

Rowena Robinson, RN, BA previously was the Project Officer with the Australian Medical Association, Canberra, Australia

Joachim P Sturmberg, MBBS, DRACOG, MFM, PhD, FRACGP is Honorary Associate Professor of General Practice at the Department of General Practice, Monash University, Australia and Conjoint Associate Professor of General Practice at the Department of General Practice, The Newcastle University, Australia

## Appendix

^1 ^**Experiences, Needs and Problems in Relation to Chronic Conditions and General Practice Care, 1993 (members of four self-help groups: diabetes, epilepsy, cancer, asthma)**. *Source: Martin C. The Care of Chronic Illness in General Practice, PhD Thesis 1998*

*Needs of people in relationship to GP and other care*

• Living with protracted uncertainty or the inevitability of decline required hope and understanding.

• Living with pain or disability or diminished social role was difficult and required empathy and encouragement.

• An awareness of the personal and social impact of illness was needed from family, friends and healthcare providers.

• Wanted the 'right GP' to provide whole-person care; characteristics of the 'right GP' included: supportive, interested, helpful in clinical and practical way, good diagnostic skills with up-to-date knowledge of when to refer, investigate and treat.

• Appropriate access and availability to GPs, specialists and other professional care when needed.

• Good communication between providers.

• Continuity of care to have condition, treatment needs known and met by someone they could trust.

*Problems*

• Difficulty finding the 'right GP' to provide whole-person care who was prepared to commit to long-term personal chronic illness care.

• GPs often lacked skills in diagnosis, willingness or ability to provide adequate explanation and information, often rejected patients when they were threatened by the incompleteness of their own skills or medical knowledge.

• The frequent inability of the GP to know enough about specific conditions, but common inability of the specialist to know the whole person.

• A lack of time given to explanation and information about medical, psychosocial and practical issues related to disease management.

• The dynamics of a long-term doctor-patient relationship usually lead to support or friendship, but could lead to complacency or oversight.

• Although self-help groups filled an important gap, understanding of the individual, their chronic illness and circumstances by professionals was often lacking.

^2 ^**Comparison and Contrast of Chronic Disease Groups Themes (1993 and 2003)**

General Practice Care

• ***Consistent themes across time and different disease groups***

∘ Finding the right GP who had the following very important positive characteristics.

▪ Personal qualities – understanding, support, trust, truthfulness.

▪ Provided adequate time and timely care in general practice.

▪ GP relationship and personal continuity of care over time.

▪ Clinical competence with disease knowledge and medication knowledge.

• ***Differences among disease groups***

∘ The Epilepsy group expressed greater reliance on the '*right specialist' *rather than the GP, in relation to diagnostic skills and disease knowledge. *Prescribing and therapeutic *issues were of concern.

∘ The Cancer group who were all in remission placed very high reliance on the GP diagnostic skills in the very fraught area of detecting early recurrence or complications.

• ***Differences across time***

∘ GPs were busier and had less time than previously.

Team/Coordination/Referral Issues

• ***Consistent themes across time and different disease groups***

∘ The 'right' GP, specialist, the pharmacist and community nurses.

∘ Team integration and communication were essential. In order to help in chronic illness, there was a need to have good communication among providers, else efforts were counterproductive.

• ***Differences among disease groups***

∘ The specialist was deemed to be central to care, particularly in relation to Epilepsy and Cancer not in remission, while the GP was main provider of diabetes and epilepsy care.

∘ The role of the pharmacist was most important for the Epilepsy group.

∘ Community nursing was highly valued, particularly by the Cancer group.

∘ Palliative care was very important for cancer care.

∘ Dying was not present as a theme for the asthma, epilepsy and diabetes groups.

• ***Differences across time***

∘ GP Care Planning/Case Conferences and team care initiatives were recognised as being very important, especially GP Care Planning, although there was little experience of these activities.

Disease/Illness Issues

• ***Consistent themes across time and different disease groups***

∘ Gaining *control *of the condition. This was the most important goal in disease management and chronic illness care, bringing health condition(s) into a life fulfilling and non-life threatening state.

∘ Community lack of knowledge of disease issues.

• ***Differences among disease groups***

∘ Research/medical understanding of disease was driven by drug company/government priorities.

∘ Epilepsy was perceived to be the least well researched and understood across the whole spectrum.

∘ Stigma and discrimination were particularly noted in relation to epilepsy and some cancers.

• ***Differences across time***

∘ Improving research, knowledge and care for asthma and diabetes. Perceptions that some cancer such as breast and bowel were much advanced while other cancers such as brain cancers were neglected. Epilepsy was perceived to be lagging further behind other diseases.

∘ Stigma and discrimination were emerging in relation to obesity and diabetes.

The 'Self'

• ***Consistent themes across time and different disease groups***

∘ Control in chronic illness. Gaining control of the self and holding on to one's identity was a strongly emergent and spontaneous theme across conditions and time.

∘ Self-knowledge of one's own disease and body. This was universally agreed to as the most important prerequisite to gaining control. These were both strong themes reflecting an 'expert patient' philosophy [41]. Age and life stage were factors that shaped self-image in chronic illness.

∘ Self-management/self-help and peer support. It is noteworthy that as these informants were all members of self-help groups that they were all supportive of the concepts.

• ***Differences among disease groups***

∘ Epilepsy comprised more members who were younger and the group formed because of personal difficulties with identity, personal development, education/employment, stage of life expectations and intrusive chronic illness.

• ***Differences across time***

∘ Personal themes were consistent over time

^3 ^An Enhanced Primary Care (EPC) GP management plan (GPMP) is a comprehensive written plan that describes: the patient's health care needs, health problems and relevant conditions; management goals with which the patient agrees; actions to be taken by the patient; treatment and services the patient is likely to need; arrangements for providing these treatment and services; a date to review these matters. Reviewing a GPMP is important.



accessed 13/1/09

^4 ^An EPC case conference is a meeting of health and care providers to plan for the health care needs of an individual with at least one chronic medical condition and complex multidisciplinary care needs requiring care from a GP and at least two other health or care providers. Case conferences may be undertaken in the community, on discharged into the community, or in Residential Aged Care Facilities. 

accessed 13/1/09

^5^The Sharing Health Care Initiative (SHCI) is designed to improve the health related quality of life for people with chronic diseases, to encourage people to use the health care system more effectively and to enhance collaboration between individuals, their families, carers and health care professionals in the management of chronic disease



^6 ^Asthma Chronic Disease management. The Asthma Cycle of Care is a tool for general practitioners (GPs) and people with moderate to severe asthma to work together to improve asthma management and quality of life through an ongoing cycle of best practice asthma management.



accessed 13/1/09
